# Development of the FORUM: a new patient and clinician reported outcome measure for forensic mental health services

**DOI:** 10.1080/1068316X.2021.1962873

**Published:** 2021-08-23

**Authors:** Howard Ryland, Jonathan Cook, Rob Ferris, Sarah Markham, Christian Sales, Raymond Fitzpatrick, Seena Fazel

**Affiliations:** a Department of Psychiatry, University of Oxford, Oxford, UK; b Nuffield Department of Orthopaedics, Rheumatology and Musculoskeletal Medicine, University of Oxford, Oxford, UK; c James Nash House Forensic Mental Health Service, Adelaide, Australia; d Institute of Psychiatry, Psychology and Neuroscience, King’s College London, London, UK; e Nottinghamshire Healthcare NHS Foundation Trust, Nottingham, UK; f Nuffield Department of Population Health, University of Oxford, Oxford, UK

**Keywords:** Outcomes, PROM, CROM, forensic, Delphi

## Abstract

Forensic mental health services provide care to people in secure psychiatric hospitals and via specialised community teams. Such services are typically low volume and high cost, often highly restrictive and average duration of inpatient care prior to discharge is long. Measuring outcomes of care is important to safeguard patients and the public, monitor progress, inform treatment plans and assist in service evaluation and planning. We describe the development in England of a new outcome measure for forensic mental health services. Patient interviews and multistakeholder focus groups were held to elicit key concepts. Thematic analysis was used to develop an outcomes framework. Fifteen patients participated in the interviews and 48 stakeholders in the focus groups. Six domains were identified in thematic analysis: ‘about me, my quality of life, my health, my safety and risk, my life skills and my progress’. Sixty-two stakeholders participated in the first round of the Delphi process, and 49 completed round two. Eight of the top fifteen outcomes were shared between patients/carers and professionals. Based on these results, a new outcome measure, the FORensic oUtcome Measure (FORUM), was developed including both a patient reported and clinician reported measure. Further assessment of the FORUM’s use to track patients’ progress over time, and facilitate shared decision-making and care planning, is required.

## Introduction

Forensic mental health services provide care to people with mental illness who have been convicted of serious crimes or deemed at high risk of violence towards others (Crocker et al., 2017). Individuals are typically treated in secure inpatient psychiatric hospitals and progress to lower levels of security, before returning to the community, where they continue treatment with specialised forensic community or general adult mental health teams (Latham & Williams, 2020). Average length of stay is long, with some patients remaining in hospital for years, or never successfully returning to the community (Völlm, 2019). Patients are often subject to considerable restrictions, especially as inpatients. The consequences of repeat offending can be severe for victims, their families and society (Fazel et al., 2016; Vollm et al., 2018). Such services can be very expensive, due to factors such as high staffing ratios and the need for specialised facilities (Centre for Mental Health, 2013). For these reasons measuring the outcomes of such services is important for patients, their family and wider society.

Although progress has been made to establish the most important outcomes in forensic mental health services the patient perspective has frequently been overlooked (Shin-kfield & Ogloff, 2015). This may be due to the heavy emphasis placed on risk assessment and reduction, and the severity of mental illness for most patients (Nilsson et al., 2009). Most existing outcome measures used in forensic mental health services are either based on theoretical models (O’Dwyer et al., 2011), empirical studies of risk or protective factors (Chambers et al., 2009), or are adaptations of instruments developed in general adult psychiatric populations (Dickens et al., 2007; Thomas et al., 2008). This means that the specific outcomes important in this population are not captured and therefore could be incorrectly prioritised by services, which rely on structured measurement instruments to quantify and record outcomes (NHS England, 2020c).

There are different perspectives on what constitutes a good outcome in forensic mental health services. The dominant view is driven by public protection, which prioritises the reduction in risk to others as the main goal (Buchanan & Grounds, 2011). More recently increasing emphasis has been placed on the recovery paradigm, as adapted to fit the forensic context (Clarke et al., 2016). Given the range and severity of needs of many forensic patients, measuring recovery is multifaceted and highly complex. It is associated with an improved quality of life and social functioning, and reduced risk to others (Bouman et al., 2009; O’ Flynn et al., 2018). Therefore an approach that seeks to reduce those risks should consider the whole spectrum of outcomes.

Recent policy initiatives have prioritised the need for greater patient involvement in outcome reporting in mental health services (NHS England and NHS Improvement, 2016). They also stress the need to pay greater attention to measuring outcomes, to ensure such services are operating as intended and providing good value care (Obbarius et al., 2017). Good content validity of instruments is key to ensure adequate coverage of the relevant domains (Terwee et al., 2018).

In this paper, we describe the development of a new instrument for measuring outcomes in forensic mental health services, which is designed to have complementary questionnaires for patients and clinicians.

## Materials and methods

**Figure 1:**
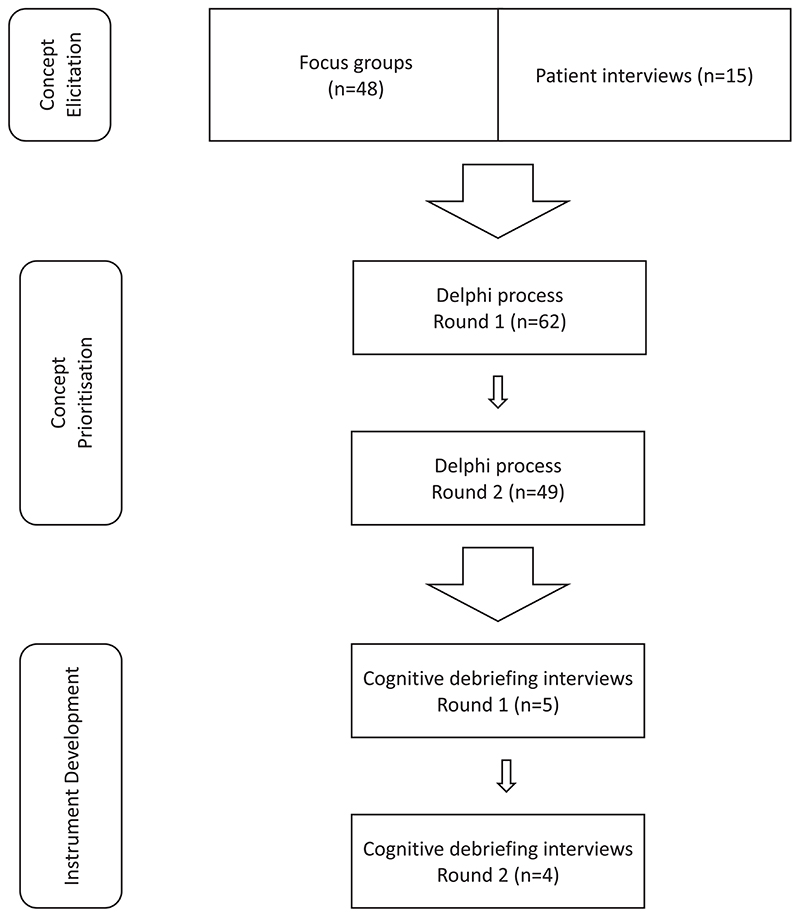
Diagrammatic representation of the three stages of concept elicitation, concept prioritisation and instrument development, including the number of participants in each stage.

The approach used to develop the new outcome measure was an iterative process involving multiple, linked components (de Vet et al., 2011). The development process had three main parts: firstly concept elicitation, secondly concept prioritisation and thirdly drafting and refinement of the new instrument. See Figure 1 for a diagrammatic representation of these stages. All parts of the work were overseen by the research team, supported by a dedicated Patient and Public Advisory Group (PPAG). One of the authors was a member of the group. The PPAG consisted of five people with lived experience of forensic mental health services, who met with the lead researcher on four occasions. Two people who were carers for patients in such services were also consulted remotely by the lead researcher, using email.

### Concept elicitation

A combination of interviews and focus groups was used to elicit concepts considered important by stakeholders. As well as ensuring the participation of a full range of the desired stakeholders, triangulation between interviews and focus groups helped to ensure stability of themes across contexts, by using different data sources to gain a more comprehensive understanding of the concepts of interest (Carter et al., 2014). Individual semi-structured interviews were conducted with patients at different stages of the forensic psychiatric pathway. Participants were purposively sampled from within Oxford Health NHS Foundation Trust and Nottingham Healthcare NHS Foundation Trust to include patients in high, medium and low security settings and in the community (Nottinghamshire Healthcare NHS foundation Trust, 2021; Oxford Health NHS Foundation Trust, 2020). Sampling also aimed to ensure that both women and men were included, given that women form only a small fraction of patients in such services and are often excluded from research (Tomlin et al., 2021; Birgit Völlm et al., 2017). Patients were initially approached by their clinical teams and invited to take part.

Focus groups were used to explore the views of multiple different stakeholders in various combinations. The sampling strategy varied depending on the context of the focus group (Etikan et al., 2016). One focus group was convened by inviting staff in forensic services at Oxford Health NHS Foundation Trust by email. The remaining focus groups were scheduled to take place after other meetings where relevant stakeholders were already gathered. The study was described to attendees at the end of these meetings, who were then invited to take part in a focus group. This approach was designed to involve a wide range of stakeholders, by ensuring that participation was as convenient as possible. A number of these meetings were conducted alongside meetings of the Adult Secure Clinical Reference Group at NHS England and the Quality Network for Forensic Mental Health Services at the Royal College of Psychiatrists (Georgiou et al., 2019; NHS England, 2020a). Both of these organisations include patients and carers in formal roles as experts by experience in their meetings.

Topic guides for the interviews and focus groups were developed in collaboration with the PPAG (see Appendix A, supplementary material). All interviews and focus groups were conducted by a trained researcher. The contents were audio recorded with the consent of the participants and professionally transcribed (Brédart et al., 2014).

Thematic analysis was used to develop themes by the lead researcher using NVivo version 12 (Castleberry & Nolen, 2018). The transcripts from the interviews and focus groups were analysed together. The emerging framework was shared with the rest of the research team and the PPAG, who provided extensive feedback. Overlapping groups of themes were amalgamated and additional groupings were added if necessary to produce the draft outcome framework. To enhance trustworthiness a second independent researcher used this framework to deductively code half of the original transcripts, to ensure that the codes covered all of the concepts in the data. A senior member of the research team with extensive qualitative research experience also reviewed one interview and one focus group transcript using the framework (Nowell et al., 2017).

### Concept prioritisation

A Delphi technique was used to obtain the views of a range of stakeholders about the outcome areas from the thematic analysis. All participants in the concept elicitation stage were invited to participate. Additional participants were invited through the Recovery and Outcomes network (Recovery and Outcomes, 2020), Oxford Health NHS Foundation Trust (Oxford Health NHS Foundation Trust, 2020) and the Adult Secure Clinical Reference Group at NHS England (NHS England, 2020a). The survey was administered either electronically via an online platform (Delphi Manager), or in paper and pen format by a researcher, for patients without internet access (Crew & Williamson, 2020). All participants were required to choose one of three stakeholder categories, namely patient, carer or professional.

In the first round, outcomes were presented under the overarching domains derived from the thematic analysis. Participants were presented with one domain at a time, containing all the outcome areas within that domain. The domains were presented in a random order to reduce the risk of bias resulting from respondent fatigue, which might otherwise affect those domains consistently presented later in the survey (Brookes et al., 2018).

Each outcome area was presented in the first person, as a simple statement, using the wording agreed by the research team and the PPAG. Additional help text was available to describe each of the outcome areas in more detail if necessary. Participants were asked to consider the importance of each area from their own perspective, depending on their role or background. They were asked to rate each outcome area on a 9-point scale (de Meyer et al., 2019).

Following the first round, participants’ answers were analysed in two stakeholder groups of (1) patients and carers and (2) professionals (MacLennan et al., 2018). Histograms of the distribution of scores were produced for each outcome area for each of the two stakeholder groups. Participants’ additional suggestions were reviewed by the research team and compared to the existing outcome areas. Any outcome area that was not already covered was added to the next round, in the relevant domain.

In the second round participants were asked to score any additional outcomes in a similar way to the first round. For the outcome areas previously included in the first round, all participants were presented with histograms of the distributions of scores in both stakeholder groupings described above. Participants were also able to view their own scores from round 1 and were then asked if they wanted to keep the score the same or alter it up or down, in light of the views of others, as presented in the histograms. The mean score for round 2 was calculated for each outcome area in each of the two stakeholder groups of (1) patients and carers and (2) clinicians.

The results of the Delphi process were presented to four multi-stakeholder groups to contextualise the results and obtain further feedback (Williamson et al., 2017).

### Development of the new instrument

The new instrument was conceptualised to contain two separate, but linked questionnaires: a patient reported outcome measure (PROM) and a clinician reported outcome measure (CROM). Ten principles guided the development process to ensure that the resultant instrument fulfils its intended purpose and mechanism of use (see Appendix B, supplementary material).

The selection of items for inclusion in the initial draft was guided by discussions in the research team, based on the results of the concept elicitation and prioritisation work. The introduction and instructions were designed to be as short and functional as possible. The timeframe for ratings was guided by the expected pace of change in forensic services. The length of stay is long in such services and comprehensive reviews usually take place every six months through the Care Programme Approach (Hare-Duke et al., 2018; Tomlin et al., 2021).

The PROM was then reviewed by the PPAG and revised before undergoing two rounds of cognitive debriefing interviews (CDI) with patients. Patients in Oxford Health NHS Foundation Trust who had participated in the concept elicitation and prioritisation exercise were invited to participate. The CDIs included a ‘thinking aloud exercise’ and a series of verbal probes (Drennan, 2003). The CDIs were informed by a topic guide developed in collaboration with the PPAG (see Appendix C, supplementary material). The PROM was revised after each round of CDIs. The CROM was also reviewed by the PPAG and clinicians from a range of professional backgrounds. The final drafts were agreed by the research team and PPAG (de Vet et al., 2011).

### Ethics approval and consent to participate

This study was approved by the London – Surrey Research Ethics Committee (Reference 18/LO/0929). All participants provided informed consent to participate.

## Results

### Concept elicitation

Fifteen interviews were conducted between 16th October 2018 and 10th December 2018. Participants comprised 2 patients from high security, 4 from medium security, 7 from low security and 2 from the community. Three out of the 15 patients were women and the remainder men. Seven focus groups comprising 48 unique individuals took place between 4th September 2018 and 17th December 2018. Participants comprised 9 psychiatrists, 8 managers, 6 psychologists, 5 nurses, 5 patients, 4 occupational therapists, 4 commissioners, 3 carers, 3 policy staff members and 1 social worker. For more details on the focus groups see Appendix D (supplementary material).

The final outcome framework contained six overarching domains containing 42 individual outcome areas. The domains were ordered according to a suggestion from the PPAG and agreed by the research team. This order moves from more personal, internal and subjective concepts, to those that are more objective and externally determined. It is not however intended to represent a hierarchy of importance. All domains and constituent outcome areas are framed in the first person to emphasise the importance of the individual patient’s perspective (Wallang et al., 2018).

The first domain, termed ‘about me’, concerns questions of self-concept and encompassed ideas of meaning and belonging, as well as feelings of control and wellbeing. The next domain ‘my quality of life’ considers the interaction of a person with the outside world and the degree of satisfaction this affords that individual. Outcome areas in this domain span from simple items, such as whether a person’s basic needs are met, to more complex concepts, such as whether they feel respected and accepted by others. ‘My health’ then considers the individual’s physical and mental health, such as whether they are able to maintain a healthy weight or their mental and emotional health is good. ‘My safety and risk’ concerns both behavioural and internalised aspects of risk, such as avoiding behaviours and situations that reduce safety and recognising and coping with difficult feelings. ‘My life skills’ incorporates both current and future capabilities, from being able to look after every day needs, to being able to make realistic plans. Finally, ‘my progress’ attempts to quantify the person’s position within the care pathway, both considering where that individual is at that point in time, ‘I am at the right place for me in the mental health system’, to thinking about where they need to move towards, ‘I am making progress towards greater independence’. The full framework is described in Table 1. For a more detailed description of each domain and the primary data to support it, see Appendix E (supplementary material).

### Concept prioritisation

The Delphi process ran between 4th June 2019 and 9th March 2020. Sixty two people participated in the first round of whom 49 went on to participate in the second round. The stakeholder groups are described in Table 2.

A total of 13 additional outcomes were suggested by 7 participants (see Appendix F, supplementary material). The research team decided that only 1 outcome, ‘I am actively working on reducing my risk of harm to others’ was not covered by concepts already in the outcome framework and should therefore be added to the second round of the Delphi process under ’my safety and risk’ (Williamson et al., 2017).

### Patients and carers

The average score overall was 7.36 for round 1 and 7.64 for round 2. ‘I feel safe’ was the top rated outcome after round 2, with an average score of 8.33. Out of the top 15 outcomes after round 2, six were from the domain ‘my health’, five were from ‘my risk and safety’, two from ‘my progress’ and one each from ‘my life skills’ and ‘my quality of life’. See Table 3 for the full results.

### Clinicians

The average total score was 6.93 for round 1 and 7.05 for round 2. ‘My basic needs are met’ was the top rated outcome after round 2, with an average score of 8.33. Out of the top 15 outcomes after round 2, seven were from the domain ‘my risk and safety’, four from ‘my health’, and one each from ‘about me’, ‘my quality of life’, ‘my life skills’ and ‘my progress’. See Table 4 for the full results.

### Development of the new instrument

The initial draft of the PROM consisted of 18 items and the CROM consisted of 21 items. Items were deliberately selected from all relevant domains to ensure comprehensiveness. Rather than simply choosing those items with the highest overall mean scores in the Delphi process, scores were used to help select items within domains. The items in the PROM were chosen from all 6 domains in the framework described in Table 1, while the CROM only included items from the final 4 domains, as ones that clinicians could meaningfully comment on. A recall period of 1 month was chosen to balance the need to be brief enough to capture change, while not being too short as to be impacted by day to day fluctuations (Clarke et al., 2008). The response options were the same for each item using a five point Likert scale based on the concept of agreement, ranging from ‘strongly agree’, to ‘strongly disagree’ (Bishop & Herron, 2015).

As a result of feedback from the PPAG, several changes were made to the PROM before it was tested in the CDIs. Two additional items were added to cover future planning and the acquisition of skills. The response options were changed from ones of agreement to ones based on frequency, ranging from ‘never’ to ‘always’. The CROM was also altered, so that the response options aligned with the revised PROM. A section was introduced to the CROM where the name of the patient being rated could be written, to increase the personalisation of the instrument and ensure a focus on that individual’s recovery (Gudjons-son et al., 2010). All the items subsequently flowed from the person’s name, for example ‘ … has had good mental health’.

Nine patients participated in the CDIs in total. Five participated in the first round between 11th and 18th November 2019. Four participated in the second round on 15th January 2020. The PROM was further revised based on the feedback from the first round of CDIs to produce the version that was tested in the second round. Further changes included expanding and elucidating the instructions, adding ‘not applicable’ to the ‘never’ response option and clarifying item descriptions. A final version was produced based on feedback from the second round, after further discussion with the PPAG. Following feedback from professional colleagues, items on physical health and substance misuse were added to the CROM (Brown et al., 2019; NHS England, 2020b). The final versions of the PROM containing 20 items and CROM containing 23 items were both approved by the research team and PPAG. These are available in Appendix G (supplementary material). This new instrument is the FORensic oUtcome Measure (FORUM).

## Discussion

### Summary of findings

We report a series of interlinked studies that aim to determine the most important outcome domains in forensic mental health services and then operationalise these in a new outcome measure. We developed a framework containing six domains. While there was some overlap in the areas prioritised by the two stakeholder groups, there were also differences. Eight out of the top fifteen outcomes were shared between patients/carers and professionals. Finally, we developed the FORensic oUtcome Measure (FORUM), a new instrument for measuring outcomes in forensic mental health services based on the concept elicitation and prioritisation studies, with additional patient input in the form of cognitive debriefing interviews (de Vet et al., 2011). These studies were informed by a patient and public advisory group, working with the research team (Grundy et al., 2019).

### Implications for policy and practice

Our outcome framework has similarities and differences to others previously advanced in forensic mental health services. The central four domains of ‘my quality of life’, ‘my health’, ‘my safety and risk’ and ‘my life skills’ map closely to those presented in previous systematic reviews (Fitzpatrick et al., 2010; Shinkfield & Ogloff, 2015). However, our framework adds the domains of ‘about me’, concerned with fundamental issues of personal identity, and ‘my progress’, which considers an individual’s pathway through mental health services. These domains were not considered in these previous reviews, which focused mainly on clinician reported outcome measures. Overall there are many similarities between our framework and other empirically derived frameworks (Wallang et al., 2018) (Livingston, 2018) (Morrissey et al., 2017). While the content of the individual items is similar to these frameworks, they use different terms to describe the outcomes and also order them differently. This is reassuring in that it demonstrates that frameworks with broadly similar domains emerge when different methods are used in other contexts (Hansen, 2020).

Many of the priorities for patients and carers differ from those considered most important by clinicians (Rose et al., 2006). Measurement of outcomes should consider areas seen as priorities for both groups of stakeholders. Informants for these outcome measures should be drawn from the stakeholder group that is best placed to comment on the relevant issue, and more than one perspective should be sought where appropriate (de Vet et al., 2011). Although risk/safety and clinical symptoms/health remain important to both stakeholder groups, participants at the Delphi feedback meetings strongly supported the idea that a stratified approach should be used when developing a new outcome measure. A stratified approach involves selecting outcomes from each of the relevant overarching domains, to allow adequate breadth of coverage and ensure a more ‘balanced scorecard’. This is consistent with guidelines, which caution against the overly simplistic use of quantitative results from Delphi surveys (Williamson et al., 2017).

The FORUM offers an approach for measuring these concepts. It consists of two singlepage questionnaires designed to be rated by patients or clinicians respectively. This could facilitate therapeutic discussions between patients and their clinical teams (Coffey et al., 2019). It may also allow patients to track their progress over time, through repeated measurements at regular intervals (Clark et al., 2018). It addresses fundamental questions of identity, quality of life, life skills and pathways that need greater attention in the commissioning and evaluation of services (Samartzis & Talias, 2020). Although the new instrument appeared to have good comprehensibility, comprehensiveness and relevance in the small sample included in the CDIs, this requires corroboration in a larger sample. Wider testing is also needed to explore the other psychometric properties of the new instrument, such as structural validity, reliability, and responsiveness (Prinsen et al., 2018). Further changes may be necessary based on these results, with subsequent testing in new samples.

The new instrument is intended to be used in practice by patients and clinicians working together towards a shared understanding. Further work is needed to explore how this can be done most effectively, including the timings of when each party completes their respective questionnaires, how scores are discussed between patients and clinicians, how responses are tracked over time and what degree of change is clinically important. Thought will also need to be given to the utility of using aggregate scores of all of the items in each questionnaire, rather than considering changes in each item individually.

### Strengths and limitations

The main strength is the use of an iterative methodology based on current best practice in outcome measure development (de Vet et al., 2011). All decisions were empirically informed and involved considerable input from the full range of stakeholders, including patients both as participants and in guiding the research through the PPAG (Wiering et al., 2017). The PROM and CROM were developed simultaneously, and so are designed to be complementary. The PROM underwent repeated testing with patients in its development, to enhance its acceptability and optimise content validity.

A number of limitations should be considered. Although efforts were made to purposively sample patients from a range of settings, we recruited from just two NHS Trusts within a single country. The gatekeeping role of clinicians in participant selection will have influenced which patients were approached to participate (Sharkey et al., 2010). It is possible that those approached were viewed as less unwell or more compliant by their treating teams, which risks excluding the views of those who were more unwell or seen as less cooperative by clinicians. The interviewer’s professional background as a psychiatrist may have influenced the information that participants were willing to share in interviews (Vale & Good, 2020).

Participation of patients in the Delphi process through a researcher could have influenced their answers, as they may have been inclined to provide answers that they thought the researcher would want. Attrition of participants between the first and second rounds may account for some of the differences in ranking of items between these two rounds, rather than an actual shift in participants’ perspective (Brady, 2015). The small range of mean scores in the Delphi process limits the power to discriminate between the outcomes.

Participants in the cognitive debriefing interviews may not have felt comfortable raising criticisms of the draft PROM with a professional researcher, due to a perceived imbalance in status, power and expertise (Birgit Völlm et al., 2017). The number of patients involved in the cognitive debriefing interviews was relatively small, although this was in line with recommended best practice (Terwee et al., 2018). The CROM underwent relatively limited testing, relying on the professional expertise within the research team and discussions with clinical colleagues.

Patients with intellectual disability were not included as participants in the development and therefore the FORUM in its current form may not be suitable for the needs of this group. Further work would be needed to assess the use of the FORUM in this population and any potential adaptation that may be required to meet their specific needs.

## Conclusions

The identification and prioritisation of important outcomes in forensic mental health services requires the involvement of the full range of stakeholders, especially patients, whose input has previously been overlooked. Instruments to measure these outcomes should be empirically based. We developed a framework of outcomes for such services, arranged under six domains. Identity and pathway are domains that have not been adequately considered previously. We present the FORUM, a new instrument, which includes both a PROM and a CROM, for measuring outcomes from across the six domains. This could be used to help patients track their progress over time, facilitate care planning, and evaluate interventions.

## Supplementary Material

Appendix A

Appendix B

Appendix C

Appendix D

Appendix E

Appendix F

Appendix G

## Figures and Tables

**Table 1:** Final outcome framework from concept elicitation work.

*About me* (1) My life feels meaningful (2) I have a sense of belonging (3) My spiritual needs are met (4) I feel confident and have good self-esteem (5) I feel positive and hopeful (6) I feel enabled to make the changes I want to achieve my goals (7) I feel a sense of independence
*My quality of life* (1) My basic needs are met (2) My life has the structure I want and need (3) I have enough opportunity for enjoyable and meaningful activities (4) I have the relationships and friendships I want and need (5) I feel accepted by others and comfortable around them
*My health* (1) I am able to sleep well (2) I eat healthily (3) I am able to maintain a healthy weight (4) I have enough exercise (5) I can manage any unhealthy habits and behaviours (6) My mental and emotional health is currently good (7) I understand and manage my mental and emotional health well (8) I have helpful relationships with staff (10) I take any medication I need for my recovery (11) Any side effects from my medication are manageable (13) I am able to benefit from talking therapies (14) I am able to participate in planning my care
*My safety and risk* (1) I can recognise and cope with difficult feelings (2) I am able to manage difficults situations well (3) I am able to avoid behaviours and situations that reduce my safety (4) I understand and accept rules that enable everyone to remain safe (5) I feel safe (6) I am able to take responsibility for myself (7) I understand how my behaviour may affect others (8) I am able to recognise and respect the feelings of others (9) Others feel safe around me
*My life skills* (1) I am able to make realistic plans (2) I can make the decisions that I need to (3) I am able to trust others and ask for help when I need it (4) I am able to look after my everyday needs and those of people who depend on me (5) I have the skills, education and qualifications that I want (6) I am able to play a part in my community
*My progress* (1) I am at the right place for me in the mental health system (2) I am receiving and accepting the support I need from mental health services (3) I am making progress towards greater independence

**Table 2:** Participants in the Delphi process by stakeholder group.

	Round 1	Round 2
Professionals	38	28
Patients	20	18
Carers	4	3
Total	62	49

**Table 3:** Results of the Delphi process for patients and carers.

Round 2 rank	Outcome	Domain	Round 1 score	Round 1 rank	Round 2 score
1	I feel safe	My risk and safety	8.04	2	8.33
2	My basic needs are met	My quality of life	7.78	6	8.33
3	I am making progress towards greater independence	My progress	7.96	4	8.29
4	I am receiving and accepting the support I need from mental health services	My progress	8.09	1	8.19
5	I am able to trust others and ask for help when I need it	My life skills	7.92	5	8.05
6	I understand how my behaviour may affect others	My risk and safety	7.67	8	8.00
7	I am actively working on reducing my risk of harm to others	My risk and safety	N/A	N/A	8.00
8	I am able to participate in planning my care	My health	8.00	3	7.95
9	My mental and emotional health is currently good	My health	7.52	15	7.95
10	Others feel safe around me	My risk and safety	7.21	28	7.95
11	I am able to benefit from talking therapies	My health	7.57	12	7.90
12	I take any medication I need for my recovery	My health	7.77	7	7.90
13	I am able to sleep well	My health	7.57	11	7.86
14	I understand and manage my mental and emotional health well	My health	7.52	16	7.86
15	I understand and accept rules that enable everyone to remain safe	My risk and safety	7.25	26	7.86
16	I am able to recognise and respect the feelings of others	My risk and safety	7.17	29	7.86
17	My life feels meaningful	About me	7.65	9	7.81
18	I am able to avoid behaviours and situations that reduce my safety	My risk and safety	7.54	13	7.81
19	I feel confident and have good self-esteem	About me	7.52	14	7.81
20	I am at the right place for me in the mental health system	My progress	7.52	17	7.81
21	I am able to take responsibility for myself	My risk and safety	7.39	20	7.81
22	I can make the decisions that I need to	My life skills	7.48	18	7.76
23	I feel positive and hopeful	About me	7.61	10	7.71
24	I can recognise and cope with difficult feelings	My risk and safety	7.46	19	7.71
25	Any side effects from my medication are manageable	My health	7.32	22	7.70
26	I am able to look after my everyday needs and those of people who depend on me	My life skills	7.38	21	7.62
27	I have enough opportunity for enjoyable and meaningful activities	My quality of life	7.26	25	7.62
28	I have helpful relationships with staff	My health	7.00	37	7.57
29	I feel a sense of independence	About me	7.13	30	7.43
30	I have a sense of belonging	About me	7.30	23	7.38
31	I am able to play a part in my community	My life skills	7.29	24	7.38
32	I feel enabled make the changes I want to achieve my goals	About me	7.04	34	7.38
33	I can manage any unhealthy habits and behaviours	My health	6.91	39	7.38
34	I have the relationships and friendships I want and need	My quality of life	7.21	27	7.33
35	I am able to manage difficult situations well	My risk and safety	7.13	32	7.33
36	My life has the structure I want and need	My quality of life	7.04	35	7.24
37	I eat healthily	My health	7.13	31	7.19
38	I have enough exercise	My health	7.00	36	7.19
39	I am able to make realistic plans	My life skills	6.92	38	7.19
40	I feel accepted by others and comfortable around them	My quality of life	7.09	33	6.90
41	I have the skills, education and qualifications that I want	My life skills	6.83	40	6.90
42	I am able to maintain a healthy weight	My health	6.61	41	6.90
43	My spiritual needs are met	About me	6.26	42	6.19

**Table 4:** Results of the Delphi process for professionals.

Round 2 rank	Outcome	Domain	Round 1 score	Round 1 rank	Round 2 score
1	My basic needs are met	My quality of life	7.95	1	8.33
2	I feel safe	My risk and safety	7.81	2	8.19
3	Any side effects from my medication are manageable	My health	7.59	3	7.96
4	I take any medication I need for my recovery	My health	7.24	14	7.85
5	I am able to avoid behaviours and situations that reduce my safety	My risk and safety	7.49	4	7.77
6	My life feels meaningful	About me	7.46	6	7.74
7	I am receiving and accepting the support I need from mental health services	My progress	7.21	15	7.62
8	I can recognise and cope with difficult feelings	My risk and safety	7.46	5	7.54
9	I am able to manage difficult situations well	My risk and safety	7.43	7	7.46
10	I understand and accept rules that enable everyone to remain safe	My risk and safety	7.27	10	7.46
11	I am able to trust others and ask for help when I need it	My life skills	7.03	22	7.41
12	I am able to take responsibility for myself	My risk and safety	7.38	8	7.38
13	I have helpful relationships with staff	My health	7.30	9	7.38
14	I understand how my behaviour may affect others	My risk and safety	7.27	11	7.38
15	My mental and emotional health is currently good	My health	7.14	18	7.38
16	I am able to participate in planning my care	My health	7.03	21	7.35
17	I am actively working on reducing my risk of harm to others	My risk and safety	N/A	N/A	7.31
18	I understand and manage my mental and emotional health well	My health	7.14	17	7.27
19	I have the relationships and friendships I want and need	My quality of life	7.16	16	7.22
20	Others feel safe around me	My risk and safety	6.97	23	7.19
21	I feel positive and hopeful	About me	7.11	19	7.19
22	I have a sense of belonging	About me	7.24	12	7.15
23	I have enough opportunity for enjoyable and meaningful activities	My quality of life	7.24	13	7.11
24	I am making progress towards greater independence	My progress	6.67	29	7.08
25	I am able to benefit from talking therapies	My health	6.73	28	7.08
26	My life has the structure I want and need	My quality of life	7.11	20	7.00
27	I am able to recognise and respect the feelings of others	My risk and safety	6.84	25	7.00
28	I am able to sleep well	My health	6.89	24	6.81
29	I feel enabled make the changes I want to achieve my goals	About me	6.62	30	6.78
30	I am at the right place for me in the mental health system	My progress	6.78	27	6.77
31	I can make the decisions that I need to	My life skills	6.59	31	6.70
32	I am able to make realistic plans	My life skills	6.54	33	6.67
33	I am able to look after my everyday needs and those of people who depend on me	My life skills	6.51	34	6.67
34	I can manage any unhealthy habits and behaviours	My health	6.41	35	6.62
35	I feel a sense of independence	About me	6.41	36	6.44
36	I feel accepted by others and comfortable around them	My quality of life	6.84	26	6.41
37	I have enough exercise	My health	6.27	38	6.35
38	I feel confident and have good self-esteem	About me	6.57	32	6.33
39	I am able to maintain a healthy weight	My health	6.27	37	6.31
40	I am able to play a part in my community	My life skills	6.08	40	6.26
41	I have the skills, education and qualifications that I want	My life skills	6.27	39	5.93
42	I eat healthily	My health	5.97	41	5.93
43	My spiritual needs are met	About me	5.76	42	5.41
