# Supplementary material for: Development of the FORUM: a new patient and clinician reported outcome measure for forensic mental health services
Source: Psychol Crime Law. Author manuscript; Available in PMC 2022 Oct 21. (PMC7613634; doi:10.1080/1068316X.2021.1962873)
Supplement: Appendix A [file EMS141024-supplement-Appendix_A.docx]

**Appendix A**

*Topic guides for interviews and focus groups*

Interviews

Example questions:

1. What are the three most important things in your life right now?
2. What would a good day look like to you?
3. What activities are important for you?
4. How can your healthcare team support you in the best way? What do you need from them to support your recovery goals?
5. How important is it to be involved in decisions about your care?
6. What support do you need from other services, such as social services?
7. What does taking medication mean to you?
8. How important are talking therapies to you?
9. How important is your physical health to you?
10. How would you be confident that your mental health is improving?
11. What would mean that you do not pose a risk to yourself or other people?
12. What would progress look like to you? What might mean you had made progress?
13. How do you know that you are making progress towards the life you want to lead?
14. Can you describe what a good quality of life would look like for you?
15. Can you describe what a good future looks like for you?

Focus groups

Example questions:

1. What does it mean for a patient in forensic services to be making progress?
2. What should the ultimate goals be for patients in forensic services?
3. What outcomes should forensic services aim to achieve?
4. How important is clinical recovery as an outcome of care?
5. How important is risk reduction as an outcome of care?
6. How important is quality of life as an outcome of care?
7. How important is rehabilitation as an outcome of care?
