# Supplementary material for: Development of the FORUM: a new patient and clinician reported outcome measure for forensic mental health services
Source: Psychol Crime Law. Author manuscript; Available in PMC 2022 Oct 21. (PMC7613634; doi:10.1080/1068316X.2021.1962873)
Supplement: Appendix B [file EMS141024-supplement-Appendix_B.docx]

**Appendix B**

*Guiding principles for the development of the FORUM*

1. The content of the instruments should be based on empirical evidence derived from population of intended use.
2. The measures should be short and quick to complete, to encourage use and minimise burden on both patients and clinical staff.
3. The questionnaires should be easy to understand.
4. The questionnaires should be simple to complete without training and the response options should be intuitive.
5. The included items should provide a comprehensiveness overview of relevant concepts.
6. There should be a balance of items across relevant outcome domains.
7. The selection of items for the patient and clinician reported questionnaires should be guided by the concepts of most importance to each group and that can be measured from that rater’s perspective.
8. Items need to be meaningfully measurable through an individual’s response in a way that adds useful information and those that are more reliably measured by other means should be avoided.
9. There should be consistency in the response options between items and across questionnaires to reduce confusion and aid comparison between the patient and clinician reported questionnaires.
10. The timeframe should be long enough to be meaningful, but short enough to support use as a repeated measure.
