# Supplementary material for: Development of the FORUM: a new patient and clinician reported outcome measure for forensic mental health services
Source: Psychol Crime Law. Author manuscript; Available in PMC 2022 Oct 21. (PMC7613634; doi:10.1080/1068316X.2021.1962873)
Supplement: Appendix C [file EMS141024-supplement-Appendix_C.docx]

**Appendix C**

*Interview schedule for cognitive debriefing interviews*

**Thinking aloud exercise**

Please complete the questionnaire, talking out loud about what is going through your mind when you fill in each section.

**Verbal probes**

Relevance

Were the questions meaningful to you?

Are these questions a good way of measuring your progress in forensic mental health services?

What did you think about the choices of answer that you were allowed?

What did you think about the period of one month? How easy was it to remember what you thought during this period of time? Would a longer or shorter period of time be better?

Comprehensiveness

This questionnaire was about your progress within forensic services. Was there anything important missing?

Comprehensibility

Did you feel like you understood what you were being asked to do when filling in the questionnaire?

What did you think about the way the questions are written? Are there any that are confusing or not clear?

Were you able to find an answer to each question from the options available?
