# Supplementary material for: Development of the FORUM: a new patient and clinician reported outcome measure for forensic mental health services
Source: Psychol Crime Law. Author manuscript; Available in PMC 2022 Oct 21. (PMC7613634; doi:10.1080/1068316X.2021.1962873)
Supplement: Appendix D [file EMS141024-supplement-Appendix_D.docx]

| Date | Context | N | Composition |
| --- | --- | --- | --- |
| 04/09/2018 | QNFMHS London | 5 | Professional/patient |
| 29/09/2018 | QNFMHS Middlesbrough | 9 | Professional/carer |
| 10/10/2018 | TVWFN Southampton | 10 | Professional |
| 17/10/2018 | QNFMHS Bristol | 3 | Professional/patient |
| 04/12/2018 | AS CRG London | 13 | Professional/patient |
| 13/12/2018 | OHNFT, Oxford | 7 | Professional |
| 17/12/2018 | QNFMHS London | 5 | Professional/patient/carer |

**Appendix D**

**Table D1**

*Details of the seven focus groups*

QNFMHS – Quality Network for Forensic Mental Health Services

TVWFN – Thames Valley and Wessex Forensic Network

AS CRG – Adult Secure Clinical Reference Group

OHNFT – Oxford Health NHS Foundation Trust
