# Supplementary material for: Development of the FORUM: a new patient and clinician reported outcome measure for forensic mental health services
Source: Psychol Crime Law. Author manuscript; Available in PMC 2022 Oct 21. (PMC7613634; doi:10.1080/1068316X.2021.1962873)
Supplement: Appendix E [file EMS141024-supplement-Appendix_E.docx]

**Appendix E**

*Evidence for outcome framework*

About me

This category was the most abstract and concerns existential questions about a person’s identity. At the apex of this category was the concept of having a life with meaning.

“*The key thing is having a meaningful life”*

*“recovery in the sense of having a meaningful life”*

Closely allied to this and viewed by many as necessary for meaning to exist was a sense of belonging, which was expressed by both patients and professionals.

*“I can belong to society or a nation or a culture or a religion or a social context”*

*“the feeling of the patient belonging within the environment that they live in”*

Spiritual need was identified as strongly important by several participants, but was not universally agreed to be something of critical value. Spirituality could be overtly religious, although not necessarily so.

*“Yes, I have a faith. I’m a Roman Catholic. I have a faith. I go to church every now and then. I read the ‘Good Book’ every now and then. I think it’s important whenever you’ve got a faith in something. It’s not everybody’s cup of tea, but it’s good to be involved with something like that in your life.”*

*“Interviewer: Ok. And you mentioned the spiritual aspect of yoga? How important is spirituality?*

*Respondent: It does a lot for me, it does a lot for me yes.”*

Confidence, self-esteem, positivity and hopefulness were commonly seen as desirable aspects of an individual’s experience of the world. These concepts were often linked together and participants also noted the negative consequences when they were absent.

*“I think self-esteem is truly important. If you don’t have much self-esteem you are either a danger to yourself or you are a danger to other people. People who have low self-esteem they will either try and find a way of getting more self-esteem by their self or by making other people feel bad. And that is what I used to do. Because I had a bad self-esteem”*

*“One of those can be self-esteem, self-value, self-expectation. If someone perceives they are not valued then they may in turn value others less and value society less because they have no concept of society valuing them, so it comes back to those core needs of feeling ... I do have value.”*

*“Just having hope knowing that life is not over is very important because it can make you to be stable, it can make you not to like kick off and just want to get angry”*

The ability to make changes and develop independence was seen as a crucial outcome by many participants. Independence was not simply the practical ability to access certain activities, but at its core a sense of freedom and orientation towards the future.

*“I gradually became more future referenced and started thinking about getting a job and living on my own and all of those sorts of things.”*

My quality of life

Quality of life was seen as being closely allied to and contingent on the fundamental internal characteristics of ‘about me’, but added several key domains related to the external environment. This moved from the abstract concepts of self-actualisation to the more prosaic basic needs at the base of the hierarchy of needs.

*“So essentially really what is needed is the basics, so people need to be able to have the wherewithal to be able to get themselves food, shelter and clothing”*

Beyond these basic needs participants identified both people and activities as essential components necessary for a good quality of life. Activities in of themselves were seen as important, combined with the need for structure within daily life.

“*A good structure. You need a good structure; you need a healthy structure. You need things that are going to aid you, you know”*

Interactions with other people were viewed as important and this encompassed intimate relationships, family, friendships and also acceptance from others around them.

“*I think a wife of my own would be a good quality of life for me.”*

*“I get to see my friends that I don’t often get to see when I didn’t have home leave. It is special time.”*

*“I think, for me, it’s about this inclusion, where it’s about connectivity”*

My health

This encompassed the highest number of individual outcome areas, with twelve in total, covering both physical and mental health. Specific aspects of physical health were sleep, weight, diet and exercise.

*“Sleep is important. I’ve suffered from insomnia at times. I enjoy my sleep and I think it’s good to get a good eight hours sleep, but not over-sleep, and not staying in bed all day.”*

“*Having a balanced diet; eating the right foods; eating your five a day, your vegetables, and cooking for yourself, not just eating junk food”*

The ability to manage unhealthy or damaging behaviours encompassed a huge range of activities, although the one that participants mentioned the most was controlling substance misuse.

*“So I think to live a life without drugs will be a better life. Drugs will make my life chaotic rather than effectual and beneficial.”*

Two components were viewed as necessary for a good outcome for mental health. Both the current state of health and the ability to manage one’s own emotional wellbeing. This required an internal capacity to identify and respond to challenges to mental wellbeing, but also a good relationship with staff to provide support.

*“I understand now what it means to be well and I have been unwell before in the past.”*

*“Maintaining wellness, yes. I think if you can keep a good relationship going with your team, then that’s important. If you start dropping off and not... disengaging with your social worker or your CPN then that’s a slippery road to becoming unwell.”*

This led on to the various components of care that help people to get well and maintain wellness. This prominently featured medication, psychological therapies and the ability to take part in developing a care plan. Often it was the combination of these that was most important.

*“I am the sort of person that medication has its place but also therapy is really important as well. The medication helps me to be able to, it is kind of a crutch when I need it and then you know the crutch isn’t so much that I can’t use the skills that I have got.”*

*“Yes because care plans are really important. They kind of, it is kind of like you know when you are on a tightrope and you have got like a netting underneath you for safety and that is what a care plan is for me. I can always fall back on it if I am in a tight situation.”*

My safety and risk

This was the second largest domain, containing nine outcome areas. Several outcome areas dealt with the themes of being able to recognise within oneself difficult feelings and manage these effectively, including situations that are challenging.

“*I think again it comes back to measures of resilience, what resilience has the patient developed? Because they will have been exposed to stress within the hospital environment 24/7. So again what they have learnt from that in terms of building resistance and whether they have capacity to deploy it to different situations in the community when they are discharged.”*

An extension of this was the ability to avoid such challenging circumstances in the first place and to act within the rules that enable everyone to remain safe.

“*Well, from the day I come in, I want to get out. So I made sure that I’ve done everything I could to stay out of trouble.”*

Safety had many components, including safety from others and from oneself. This included the risk of deliberately harming oneself or behaving in such a way towards other that one’s own safety is threatened.

“*I go for suicide when things are really bad and at the moment things are going all right and I just hope the best but I just, I kind of, I feel safe sometimes but sometimes I don’t.”*

*“It’s about safety whilst in the services, safety and effectiveness of what we’re doing.”*

*“Yes feeling safe, knowing that there is someone who is actually watching over you making sure that no one is going to hurt you, you know?”*

The concept of responsibility was highlighted by many participants, which had many facets, including risk, behaviour and treatment.

*“I think responsibility is key for that as well, so the individual takes ownership over their own care, rather than it being prescribed necessarily to them as to this is what we expect. It should be a collaborative approach so they will be involved in that and they have the confidence to do it as well.”*

*“The ideal outcome would be that somebody understands their risk and has taken responsibility for managing that, whatever that might mean for them.”*

Finally, several themes emerged around the impact of behaviour on others, including the ability to understand the feelings of other and for others to feel safe around you.

*“I don’t think they involve carers at all. In twelve years, I’ve never been asked do you feel safe with your son?”*

My life skills

This encompassed a range of skills, from the ability to take decisions to making plans that are realistic and effective in order to achieve goals.

*“It is very important to know where you are going and heading. And knowing a time frame is more important as well instead of being left open ended.”*

*“I think responsibility is key for that as well, so the individual takes ownership over their own care, rather than it being prescribed necessarily to them as to this is what we expect. It should be a collaborative approach so they will be involved in that and they have the confidence to do it as well.”*

Also important was the ability to trust others and ask them for help appropriately.

*“I was this ravenous wolf that was prowling around and if anyone even looked at me in the wrong way I would give them a fat lip. So being in hospital they kind of allow you to learn to trust them in a kind of therapeutic place where you are able to, to feel comfortable. “*

Specific skills could be considered in two categories. Firstly, the practical everyday skills needed to look after oneself and those people around you, especially those who are dependent on you. Secondly, more discrete skills gained through education, including formal qualifications.

*“I have done life skills groups where we did cooking, things like that. We talked about budgeting and just loads of different stuff around life skills. I have done a talking group which was a group of patients and two members of staff and we talk about feelings and emotions, what is going on for us at that time. So, the therapy is just as important as medication, I think. Really important.”*

*“…at the end of the day you have to have some sort of responsibility because you owe it to that baby, you bring it to the world and it is yours. And you created it inside you and you should look after it, I think that is how I see it.”*

*“And finishing school, that would be a good future for me. A very good future.”*

Finally, the ability to participate in some form of community was identified as a key outcome. There was a recognition that what defined community varied widely between contexts. It could be within a ward in an inpatient environment, a specific community, such as a religious or cultural one, or in the wider society.

*“I guess as services we need to be better at helping people establish those kinds of roles and build up that kind of activity and that kind of autonomy in the community before they’re discharged.”*

*“Looking after animals. Working in a team. And it has been really important to me and it has been part of our rehabilitation getting us back in the community.”*

My progress

The last domain in the framework concerned the individual’s current position and future trajectory within forensic mental health services. This location within the hierarchy of security tiers was deemed to be fundamental to patients’ progress. The first two outcome areas focused on the actual circumstances of that person, including both whether they are in the most appropriate place within the system at that time and if they are receiving the support that they need to move forward.

*“I think as a society we are not great at enabling individuals to be able to progress. There are so many registers and restrictions and different things that are going on, which I understand the purpose for and I am not saying that perhaps they should not be there, but I think we need to realise the impact that has on the individual and how that can stop someone from being able to move on.”*

*“I don’t want to be lost in the system whereby by the time I am 30 I am not able to achieve my goals.”*

*“Yes, supporting me to stay well. I talk to one or two of them, with the nurses, with my key nurse, we talk about the future and the past and they support me to be a good person. And that’s what I’m thinking.”*

The final outcome area for the progress domain looked to the future and asked whether someone is making progress towards greater independence. There was a recognition that what constitutes independence varied according to both the context and the individual.

*“It is just going to Lambourn House and obviously trying to stay well and to manage the more responsibility that I have. Further on from that, it would be to go to supported housing, somewhere in Oxford.”*

*“It’s hugely different levels or someone attending an appointment with a therapist or the one to one session that they’ve got booked in with their key nurse up to them taking that ownership and that kind of agency with developing more independence in the community. What that person is capable of doing and what’s right for them.”*
