# Supplementary material for: Development of the FORUM: a new patient and clinician reported outcome measure for forensic mental health services
Source: Psychol Crime Law. Author manuscript; Available in PMC 2022 Oct 21. (PMC7613634; doi:10.1080/1068316X.2021.1962873)
Supplement: Appendix F [file EMS141024-supplement-Appendix_F.docx]

**Appendix F**

**Table F1**

*Additional outcome areas suggested by participants in round 1 of the Delphi process*

| **Outcome** |
| --- |
| 1. Maintaining relationships with my family members |
| 1. Family members are encouraged to be involved in my care from admission to discharge |
| 1. My family is treated with respect and has on going emotional support from the service |
| 1. Family members are invited to ward rounds; CPA meetings; tribunals and events |
| 1. I feel trusted by staff |
| 1. I am able to communicate my needs |
| 1. I feel responsible for my past offending actions |
| 1. **I am actively working on reducing my risk of harm to others** |
| 1. I am in control of my alcohol use |
| 1. I am in control of my drug use |
| 1. I want to understand my illness and symptoms of relapse |
| 1. It’s important for me to have access to exercise and physical activity opportunities |
| 1. I have enough support to live the life I want to lead |

Note: Highlighted outcome area included in Delphi process second round
