# Supplementary material for: Development of the FORUM: a new patient and clinician reported outcome measure for forensic mental health services
Source: Psychol Crime Law. Author manuscript; Available in PMC 2022 Oct 21. (PMC7613634; doi:10.1080/1068316X.2021.1962873)
Supplement: Appendix G [file EMS141024-supplement-Appendix_G.pdf]

Appendix G

FORensic oUtcome Measure (FORUM)– Patient Reported (P)

This questionnaire is designed to help us support you in your care and to aid your recovery. You can use it to help plan your care with your clinical team. It may also help you to keep track of how you feel about your progress over time. There are no right or wrong answers. Please try to complete every question if you can. If in doubt, choose the answer that feels right for you.

Thinking about yourself over the **LAST MONTH**:

	Never/not applicable	Rarely	Sometimes	Often	Always
I have felt good about myself					
I have felt hopeful					
My life has felt meaningful					
I have taken part in activities I have found enjoyable					
I have had the relationships with others I have wanted (friends, family, significant others, staff)					
I have been actively involved in planning my care					
My mental and emotional health has been good					
I have been helped by talking therapies					
I have found taking my medication helpful					
I have slept well					
I have felt safe					
I have been aware of how my behaviour could affect others					
I have been actively working on reducing my risk of harm to others					
I have been able to trust others					
I have been able to ask for help when I have needed it					
I have been able to take decisions when I have needed to					
I have been able to make the plans that I have wanted to					
I have had the opportunity to develop the skills I want					
I have made progress towards greater independence					
I have made use of the support offered by mental health services					

DRAFT

Thank you for completing this questionnaire.

© Copyright, University of Oxford, 2021. All Rights Reserved.

FORensic oUtcome Measure (FORUM) – Clinician Reported (C)

This questionnaire is designed to help you to support the recovery of your patient and allow you to track their progress over time with them. Please answer the questions using your own understanding of your patient, informed by recent multidisciplinary team discussions. Please try to answer all of the questions. If you are unsure about which answer to choose, please choose the response that first comes to mind.

Thinking about your patient over the **LAST MONTH**:

Patient's name:…………………………………………………………	Never/not applicable	Rarely	Sometimes	Often	Always
…has had good mental health					
…has had a good understanding of their mental health					
…has had good physical health					
…has taken their prescribed medication					
…has managed any side effects from their medication					
…has benefitted from talking therapies					
…appears to have slept a good amount (not too little or too much)					
…has avoided misuse of substances (alcohol and drugs)					
…has avoided behaviours that reduce their safety					
…has managed difficult situations well					
…has accepted rules that enable everyone to remain safe					
…has actively worked on reducing their risk of harm to others					
Others feel safe around them					
…has had positive relationships with staff					
…has had positive relationships with other people					
…has taken part in activities					
…has actively participated in planning their care					
…has appeared confident, with good self-esteem					
…has appeared able to trust others					
…has asked for help when needed					
…has taken the decisions they have needed to					
…has made progress towards greater independence					
…has made use of the support offered by mental health services					

DRAFT

Thank you for completing this questionnaire.

© Copyright, University of Oxford, 2021. All Rights Reserved.
